# QTL mapping for flag leaf-related traits and genetic effect of *QFLW-6A* on flag leaf width using two related introgression line populations in wheat

**DOI:** 10.1371/journal.pone.0229912

**Published:** 2020-03-19

**Authors:** Xue Yan, Shuguang Wang, Bin Yang, Wenjun Zhang, Yaping Cao, Yugang Shi, Daizhen Sun, Ruilian Jing

**Affiliations:** 1 College of Agronomy, Shanxi Agricultural University, Taigu, Shanxi, China; 2 Wheat Research Institute, Shanxi Academy of Agricultural Sciences, Linfen, Shanxi, China; 3 Chinese Academy of Agricultural Sciences, Institute of Crop Science, Beijing, China; Murdoch University, AUSTRALIA

## Abstract

The flag leaf is the main organ of photosynthesis during grain-filling period of wheat, and flag leaf-related traits affect plant morphology and yield potential. In this study, two BC_3_F_6_ introgression line (IL) populations derived from the common recipient parent Lumai 14 with Jing 411 and Shaanhan 8675, respectively, were used to map quantitative trait loci (QTL) for flag leaf length (FLL), flag leaf width (FLW), flag leaf area (FLA) and chlorophyll content (CC) at flowering stage and 15 and 20 days after anthesis (DAA) in 2016–2017 (E1) and 2017–2018 (E2) two environments. A total of 14 and 15 QTLs for flag leaf-related traits were detected in Lumai 14 / Jing 411 and Lumai 14 / Shaanhan 8675 populations, respectively. Among them, Both *QFLW-6A* and *QFLA-6A* were detected in Lumai 14 / Jing 411 population under E2 and in Lumai 14 / Shaanhan 8675 population under E1 and E2 environments, respectively. *QCC*_*S2*_*-3A* from Lumai 14 / Jing 411 population and *QCC*_*S3*_*-1A*, *QFLL-4A* and *QFLL-6A* from Lumai 14 / Shaanhan 8675 population were repeatedly identified under two tested environments. Moreover, eight QTL clusters controlling flag leaf-related traits were identified, which provided a genetic basis for significant correlations in phenotype among these traits. On the other hand, positive alleles of *QFLW-6A* for FLW detected in two populations were derived from their donors. Eighteen lines and 44 lines carried this QTL were found in Lumai 14 / Jing 411 and Lumai 14 / Shaanhan 8675 populations, respectively. The means of FLW in these lines were wider than that of the recipient parent, Lumai 14, in two environments, suggesting that *QFLW-6A* played an important role for increasing FLW. The IL 124 in Lumai 14 / Jing 411 population and the IL 59 and IL 127 in Lumai 14 / Shaanhan 8675 population had five, five and four donor chromosomal segments which carried no other QTL controlling FLW than *QFLW-6A*, respectively. And the FLWs of these lines were significantly greater than that of Lumai 14 under two environments. So these lines and their donor parent can be regarded as potential near-isogenic lines. Further, a synteny analysis found *QFLW-6A* was near the 574,283,851–574,283,613 bp fragment on chromosome 6A and 10 genes were in the range of 500 kb upstream and downstream of the fragment. These results provide the basis for identification of candidate gene and map-based cloning and functional verification of the QTL.

## Introduction

Wheat (*Triticum aestivum* L.) is a staple food crop for more than 35% of the population all over the world. The formation of grain yield in wheat is a complex physiological and biochemical process, which is related to the accumulation and assimilation of photosynthetic products during grain-filling period [1], while they are related to the function of leaves [2]. The flag leaves of wheat are considered as the main source of carbohydrates in grains, which contributed up to 50% photosynthetic activity, and about 41–43% of carbohydrates for grain filling after anthesis [3, 4]. Duwayri [5] considered that grain yield and grain number per spike decreased when flag leaves were removed. Many studies have shown that flag leaf size of wheat was positively correlated with thousand-grain weight, grain number per spike, yield per plant and other yield-related traits in cereals [6–10]. The longer the flag leaves maintain high chlorophyll content and photosynthesis, the stronger the assimilation ability of canopy, which provide more assimilation substances for grain filling, delay leaf senescence and ultimately increase grain yield [11–13]. Therefore, the flag leaf size and chlorophyll content are the main factors determining the yield potential of wheat [14–16], and optimal flag leaf size can improve photosynthesis and increase grain yield.

Flag leaf-related traits including chlorophyll content (CC), flag leaf length (FLL), flag leaf width (FLW) and flag leaf area (FLA) are all quantitative traits, and easily affected by environments. With the application of molecular marker and genetic map in crop breeding, many researchers have devoted to quantitative trait loci (QTL) mapping for flag leaf-related traits in rice [17–19], barley [20, 21], sorghum [22] and durum wheat [4, 23]. The *qFL1* for FLL and *qFLW4* and *qFSR4* for FLW have been fine-mapped, and even two genes related to FLW, *Nal1* and *Nal7* have been cloned in rice [24–28]. In wheat, QTLs controlling flag leaf-related traits have been identified on almost all 21 chromosomes [16, 29–31]. For example, using recombinant inbred line (RIL) population, 12 QTLs for chlorophyll content-related traits were mapped on chromosomes 1A, 3A, 3B, 3D, 4A, 5A, 6A, 6D, 7A and 7D [32]. Using different RIL populations, Yang et al. [33] reported five additive QTLs for CC, among them, *QChl-5A*.*1* was detected in multiple stages. Twenty-eight QTLs for CC were identified on chromosomes 1B, 2A, 2B, 2D, 3A, 3B, 4B, 4D, 5A, 6B, 6D and 7A by Shi et al. using double haploid (DH) population [34]. Using RIL population with an integrated high-density simple sequence repeat (SSR) and single-nucleotide polymorphism (SNP) genetic linkage map, 61 QTLs for flag leaf morphology trait were detected [9]. A total of 34 QTLs for flag leaf morphology trait were mapped under eight environments using RIL population, among them, two QTLs for FLW *qFlw-4B*.*3* and *qFlw-6B*.*2* and one QTL for FLA *qFla-5B* detected under more seven environments were stable QTLs [10]. Liu et al. [8] also identified 23 QTLs for FLL, FLW, FLA and flag leaf angle (FLANG) using RIL population, and four QTLs for FLL, two QTLs for FLW, four QTLs for FLA and five QTLs for FLANG were detected at least two environments. Furthermore, a major QTL for FLW, *TaFLW1*, was fine mapped at 0.2 centiMorgan (cM) interval in the 5AL12-0.35–0.57 deletion bin, which was closely linked with *Fhb5* [35]. However, these QTLs were detected using DH or RIL populations. The results were affected by both the genetic background and environment, which was difficult to be applied to breeding program. Introgression lines (IL), also known as chromosomal segment substitution lines (CSSL), are constructed by transferring chromosomal fragment from donor parent into receptor by multiple generations of backcrossing and self-crossing coupled with molecular marker-assisted selection [36]. Mapping QTL using IL population can reduce the influence of genetic background and improve the accuracy of QTL.

In this study, QTL for FLL, FLW, FLA and CC were mapped using Lumai 14 / Jing 411 and Lumai 14/ Shaanhan 8675 BC_3_F_6_ populations. The objectives of this study were to (1) identify stably expressed QTLs for flag leaf-related traits in two IL populations, (2) analyze the genetic effects of QTLs that were detected repeatedly in two populations under different environments, (3) detect potential near-isogenic lines controlling flag leaf-related traits. The propose is to provide a foundation for further fine mapping and map-based cloning.

## Materials and methods

### Plant materials and field trials

Two related BC3F6 IL populations were used in the present study, which were obtained from crossing the common receptor parent Lumai 14 with Jing 411 and Shaanhan 8675, respectively. Both IL populations contained 160 lines. Lumai 14, a variety with high grain yield potential, which was developed by the Yantai Academy of Agricultural Sciences, Shandong, China, was widely cultivated under irrigated condition [37, 38]. The donor parent, Jing 411, with strong cold resistance had been widely grown as one of the main varieties at the Northern Winter Wheat Region of China in the 1990s [39]. The other donor parent, Shaanhan 8675, was a drought-resistant and high-yield cultivar and was released in 1996 by Shaanxi Wheat Research Center, China.

Field trials were conducted at experimental station of Shanxi Agricultural University, Taigu, China (37°25′N, 112°35′E) during 2016–2017 (E1) and 2017–2018 (E2) crop seasons. All the trials were performed in randomized complete block design with three replications. The ILs together with their parents were grown in 2.5 m rows spaced 25 cm apart. Fifty seeds were sown in each row. All of the trials were irrigated before sowing. Plants only relied on natural precipitation during the whole growing period after sowing. The rainfalls in E1 and E2 growing seasons were 138.0 and 196.8 mm (http://data.cma.cn/), respectively. All field experiments were employed in accordance with standard local practices.

### Phenotyping and statistical analysis

Five plants with flowering at the same day and developing normally were randomly selected and tagged from the middle of each row. Chlorophyll content (CC) of flag leaves of the tagged plants was measured with a handheld portable chlorophyll meter (SPAD-502, Konica-Minolta, Tokyo) at flowering stage, 15 and 20 days after anthesis (DAA). The flowering stage, 15 and 20 DAA were denoted S1, S2 and S3, respectively. The reading was taken from the average of the base, middle and apical of the flag leaf. The average of CC from five plants was used as phenotypic value for each line. At S1, FLL and FLW were evaluated. Trait means of the five tagged samples from each row were used in the data analysis based on three replications. FLL was measured as the distance from the base to the tip of the leaf. The FLW measurement was taken at the widest part of the flag leaf. FLA, a derived trait, was defined as FLL × FLW × 0.75 [8, 40]. Basic statistics and Pearson’s correlation analysis among FLL, FLW, FLA and CC were performed using SPSS 20.0 (SPSS, Chicago, MI, USA).

### QTL analysis

The linkage maps of Lumai 14 / Jing 411 and Lumai 14 / Shaanhan 8675 BC_3_F_6_ populations were constructed, based on 156 and 185 polymorphic simple sequence repeat (SSR) markers, respectively [36, 41]. QTL analysis for flag leaf-related traits was performed by IciMapping 4.0 software (http://www.isbreeding.net/) with the likelihood ratio test based on stepwise regression (RSTEP-LRT). The threshold LOD values were calculated using 1,000 permutations with a type 1 error of 0.05. The QTL nomenclature was according to the rule “QTL + trait + chromosome” formula [34, 42].

## Results

### Phenotypic variation of flag leaf-related traits

FLL and FLA of both donor parents, Jing 411 and Shaanhan 8675, were much higher than those of recipient parent, Lumai 14, in all environments, and there was significant difference for FLL between Jing 411 and Shaanhan 8675 and Lumai 14, respectively. While, FLW of Jing 411 and Shaanhan 8675 was lower than that of Lumai 14, respectively. Jing 411 and Shaanhan 8675 consistently showed higher values of CC than Lumai 14 in the whole filling grain stage (Table 1). The means of FLL and CC for both IL populations in all environments were intermediate between their parents, except for CC_S1_ in E1, respectively. In both IL populations, bidirectional transgressive segregation was observed for all tested traits, showing wide phenotypic variability with the coefficients of variation (CV) ranging from 3.35 to 29.61%. The skewness and kurtosis for all treatments were less than 1.00, with the exception of CC_S2_ for Lumai 14 / Shaanhan 8675 population in E2, indicating that they were continuous variation and quantitative genetic basis.

**Table 1:** Phenotypic values for flag leaf-related traits in two IL wheat populations and their parents under two environments.

Trait^1^	Environment^2^	Parent			Lumai 14 / Jing 411					Lumai 14 / Shaanhan 8675				
		Lumai 14	Jing 411	Shaanhan 8675	Mean±SD^3^	Variation	Skewness	Kurtosis	CV(%)^4^	Mean±SD	Variation	Skewness	Kurtosis	CV(%)
FLL/cm	E1	15.13	17.12**	18.25**	15.45±1.03	13.93–17.97	0.45	0.41	6.64	15.92±1.53	12.49–18.42	-0.12	0.05	9.62
	E2	11.83	13.93**	14.26**	11.87±1.17	9.69–14.21	0.19	-0.30	9.88	11.87±1.35	9.72–14.42	0.19	-0.10	11.38
FLW/cm	E1	1.47	1.42	1.31**	1.46±0.08	1.28–1.59	-0.14	-0.26	5.61	1.54±0.10	1.31–1.79	0.16	0.31	6.52
	E2	1.31	1.27	1.18*	1.30±0.09	1.13–1.48	-0.09	0.01	6.56	1.33±0.10	1.14–1.51	-0.06	-0.20	7.17
FLA/cm^2^	E1	16.72	18.22*	17.98	16.89±1.73	14.34–20.33	0.34	0.25	10.24	18.47±2.62	14.48–23.55	0.10	-0.10	14.19
	E2	11.66	13.29**	12.62	11.62±1.70	8.26–15.76	0.32	0.00	14.66	11.94±2.00	8.85–15.89	0.10	-0.10	16.73
CC_S1_	E1	59.83	57.55	53.69*	60.34±2.02	56.15–63.97	-0.01	-0.26	3.35	60.04±2.50	52.09–64.45	-0.63	0.59	4.17
	E2	63.48	59.07**	55.43**	62.70±2.78	56.93–67.19	0.04	-0.16	4.44	63.22±3.06	54.11–67.84	-0.15	-0.02	4.84
CC_S2_	E1	59.31	55.23	52.94**	56.81±4.21	45.79–62.72	-0.95	1.39	7.41	58.97±2.64	51.21–63.84	-0.87	1.19	4.47
	E2	61.52	56.14*	54.12**	58.01±3.75	49.93–63.06	-0.67	0.90	6.46	60.39±2.75	52.90–64.57	-0.42	0.18	4.55
CC_S3_	E1	41.42	30.94*	32.75*	35.17±10.41	14.51–56.70	-0.03	-0.57	29.61	38.71±8.92	21.65–56.60	0.11	-0.63	23.04
	E2	46.61	33.42**	37.14*	35.19±9.07	16.07–54.07	-0.03	-0.49	25.78	39.39±7.61	22.80–55.84	-0.06	-0.28	19.30

^1^: FLL, flag leaf length; FLW, flag leaf width; FLA, flag leaf area; CC_S1_, chlorophyll content at flowering stage; CC_S2_, chlorophyll content at 15 days after anthesis; CC_S3_, chlorophyll content at 20 days after anthesis

^2^: E1: 2016–2017; E2: 2017–2018

^3^: SD: standard deviation

^4^: CV: coefficient of variation

* and ** indicate significant difference at the 0.05 and 0.01 probability level, respectively

### Correlation analysis for flag leaf-related traits

Significantly positive correlations were observed between FLL, FLW and FLA for both IL populations in all experiments. The correlation coefficients between FLL and FLA (r = 0.80 to 0.94) were higher than those between FLW and FLA (r = 0.76 to 0.83), which implied that FLL may be the main contributor to affect FLA. FLW was significantly positive correlated with CC at different stages after anthesis, respectively, except CC_S3_ for Lumai 14 / Jing 411 population and CC_S1_ and CC_S2_ for Lumai 14 / Shaanhan 8675 population in E2. FLA showed a highly significant positive correlation with CC_S1_ and CC_S2_ for both IL populations in E1 and CC_S3_ for Lumai 14 / Shaanhan 8675 population in E2. In addition, CC_S1_ had significantly positive with CC_S2_ and CC_S3_ in all environments, except CC_S3_ for Lumai 14 / Shaanhan 8675 population in E2. There was strongly significantly positive correlation between CC_S2_ and CC_S3_ (Table 2).

**Table 2:** Correlation coefficients for flag leaf-related traits in two IL wheat populations under two environments.

Population	Trait	FLL	FLW	FLA	CC_S1_	CC_S2_	CC_S3_
Lumai 14 / Jing 411	FLL	1.00	0.55**	0.94**	-0.13	0.06	0.11
	FLW	0.21**	1.00	0.80**	0.18*	0.23**	0.11
	FLA	0.80**	0.76**	1.00	-0.01	0.13	0.13
	CC_S1_	-0.08	0.54**	0.28**	1.00	0.44**	0.27**
	CC_S2_	-0.14	0.49**	0.21**	0.67**	1.00	0.46**
	CC_S3_	-0.29**	0.42**	0.09	0.57**	0.64**	1.00
Lumai 14 / Shaanhan 8675	FLL	1.00	0.58**	0.93**	-0.03	0.03	0.41**
	FLW	0.45**	1.00	0.83**	0.14	0.03	0.20*
	FLA	0.89**	0.80**	1.00	0.04	0.03	0.36**
	CC_S1_	0.11	0.14	0.17*	1.00	0.62**	0.14
	CC_S2_	0.09	0.22**	0.19*	0.72**	1.00	0.24**
	CC_S3_	0.10	0.17*	0.15	0.34**	0.43**	1.00

FLL, flag leaf length; FLW, flag leaf width; FLA, flag leaf area; CC_S1_, chlorophyll content at flowering stage; CC_S2_, chlorophyll content at 15 days after anthesis; CC_S3_, chlorophyll content at 20 days after anthesis

Correlation coefficients at the lower and upper triangle part are for 2016–2017 (E1) and 2017–2018 (E2), respectively

* and ** indicate significant difference at the 0.05 and 0.01 level, respectively

### Additive QTL analysis for flag leaf-related traits

**Fig 1:**
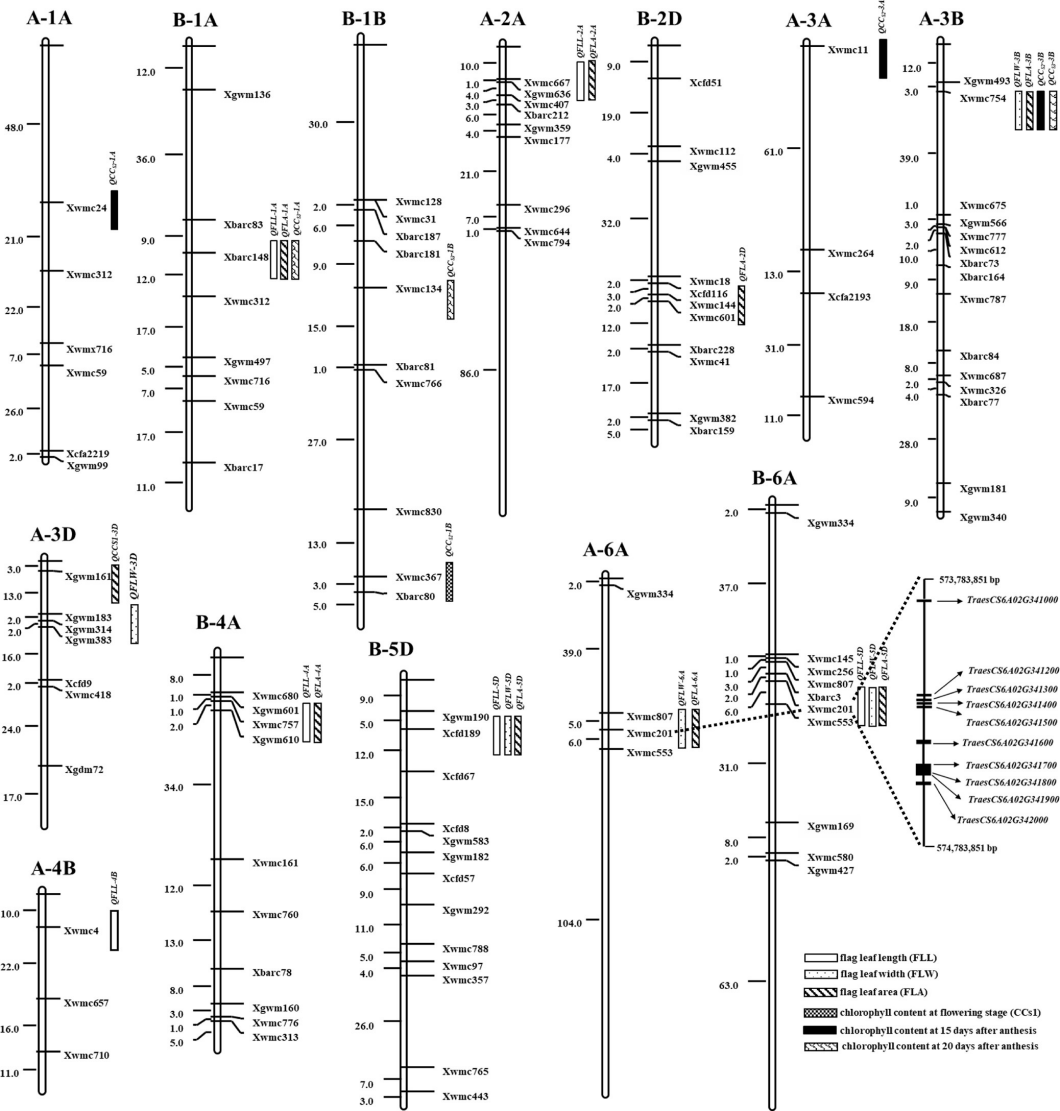
Distribution of QTLs for flag leaf-related traits on the genetic linkage map. The map distance in cM are shown on the left. The QTLs are listed on the right. A and B indicate Lumai 14 / Jing 411 and Lumai 14 / Shaanhan 8675 populations, respectively.

Fourteen additive QTLs for flag leaf-related traits were detected in Lumai 14 / Jing 411 IL population under two environments, including two QTLs for FLL, three QTLs for FLW, four QTLs for FLA, one QTL for CC_S1_, three QTLs for CC_S2_ and one QTL for CC_S3_. These QTLs were distributed on chromosomes 1A, 2A, 3A, 6A, 3B, 4B and 3D with individual QTL contributing 3.02–7.21% to the phenotypic variance (Table 3 and Fig 1). Among them, the favorable alleles of ten QTLs detected were contributed from the donor parent Jing 411, while the favorable alleles of the rest four QTLs mapped were derived from recipient Lumai 14. *QCC*_*S2*_*-3A* was detected across two tested environments, with explaining 4.09 and 3.96% of the phenotypic variance. And the locus had a favorable allele from Lumai 14 for increasing CC. The rest QTLs were detected only in one environment. In addition, four QTL clusters for flag leaf-related traits were identified. Two QTL clusters controlling FLL (*QFLL-2A* and *QFLL-4B*) and FLA (*QFLA-2A* and *QFLA-4B*) were detected on chromosomes 2A and 4B. *QFLW-6A* and *QFLA-6A* was co-located on chromosome 6A based on marker *Xwmc201*. Moreover, *QFLW-3B* was clustered with *QFLA-3B*, *QCC*_*S2*_*-3B* and *QCC*_*S3*_*-3B* with marker *Xwmc754*. The favorable alleles of these QTL clusters were derived from Jing 411.

**Table 3:** QTLs for flag leaf-related traits detected in two IL wheat populations under two environments.

Population	Trait^1^	QTL^2^	Environment^3^	Marker	LOD^4^	Add^5^	PVE(%)^6^
Lumai 14 / Jing 411	FLL	*QFLL-4B*	E1	*Xwmc47*	3.90	0.33	5.12
		*QFLL-2A*	E2	*Xwmc667*	3.37	0.41	6.38
	FLW	*QFLW-3B*	E1	*Xwmc754*	5.18	0.03	8.43
		*QFLW-3D*	E2	*Xgwm314*	2.85	-0.03	3.53
		*QFLW-6A*	E2	*Xwmc201*	4.52	0.03	6.96
	FLA	*QFLA-3B*	E1	*Xwmc754*	3.00	0.47	3.66
		*QFLA-4B*	E1	*Xwmc47*	2.55	0.43	3.16
		*QFLA-2A*	E2	*Xwmc667*	3.77	0.57	5.79
		*QFLA-6A*	E2	*Xwmc201*	3.03	0.58	5.28
	CC_S1_	*QCC*_*S1*_*-3D*	E2	*Xgwm161*	2.83	-0.68	3.02
	CC_S2_	*QCC*_*S2*_*-1A*	E1	*Xwmc24*	4.31	-2.70	4.86
		*QCC*_*S2*_*-3A*	E1	*Xwmc11*	3.66	-1.55	4.09
			E2	*Xwmc11*	3.01	-0.94	3.96
		*QCC*_*S2*_*-3B*	E2	*Xwmc754*	2.78	1.04	3.63
	CC_S3_	*QCC*_*S3*_*-3B*	E2	*Xwmc754*	3.91	3.54	4.10
Lumai 14 / Shaanhan 8675	FLL	*QFLL-4A*	E1	*Xwmc757*	7.47	-0.67	9.29
			E2	*Xwmc757*	3.25	-0.37	3.70
		*QFLL-6A*	E1	*Xwmc201*	7.95	0.54	10.08
			E2	*Xwmc201*	4.92	0.36	5.84
		*QFLL-1A*	E2	*Xbarc148*	3.32	-0.40	3.79
		*QFLL-5D*	E2	*Xcfd189*	3.64	0.35	4.32
	FLW	*QFLW-1A*	E1	*Xwmc312*	2.92	0.03	4.81
		*QFLW-6A*	E1	*Xwmc201*	5.39	0.03	9.28
			E2	*Xwmc201*	4.83	0.03	7.41
		*QFLW-5D*	E2	*Xcfd189*	3.58	0.03	5.34
	FLA	*QFLA-4A*	E1	*Xwmc757*	4.08	-0.84	5.05
		*QFLA-6A*	E1	*Xwmc201*	9.73	1.07	13.32
			E2	*Xwmc201*	5.35	0.55	6.18
		*QFLA-1A*	E2	*Xbarc148*	3.04	-0.55	3.32
		*QFLA-2D*	E2	*Xwmc144*	2.53	-0.53	2.78
		*QFLA-5D*	E2	*Xcfd189*	5.44	0.62	6.34
	CC_S1_	*QCC*_*S1*_*-1B*	E2	*Xwmc367*	3.97	-2.63	5.08
	CC_S3_	*QCC*_*S3*_*-1B*	E1	*Xwmc134*	3.55	2.51	7.38
		*QCC*_*S3*_*-1A*	E1	*Xbarc148*	5.76	-4.72	12.19
			E2	*Xbarc148*	3.32	-3.17	7.46

^1^: FLL, flag leaf length; FLW, flag leaf width; FLA, flag leaf area; CC_S1_, chlorophyll content at flowering stage; CC_S2_, chlorophyll content at 15 days after anthesis; CC_S3_, chlorophyll content at 20 days after anthesis

^2^: QTL, quantitative trait locus

^3^: E1, 2016–2017, E2, 2017–2018

^4^: LOD, logarithm of the odds

^5^: Add, additive effect, positive and negative values indicate that phenotypic variation are contributed by recipient parent and donor parent, respectively

^6^: PVE, phenotypic variation explained

In Lumai 14 / Shaanhan 8675 IL population, a total of 15 QTLs controlling flag leaf-related traits were mapped on chromosomes 1A, 4A, 6A, 1B, 2D and 5D in all experiments, seven of which carried the favorable alleles from Lumai 14 (Table 3 and Fig 1). There were four, three, five, one and two QTLs for FLL, FLW, FLW, CC_S1_ and CC_S3_, respectively, with the phenotypic variation ranging from 2.81 to 14.79%. Of them, five QTLs, *QFLL-4A*, *QFLL-6A*, *QFLW-6A*, *QFLA-6A* and *QCC*_*S3*_*-1A*, were repeatedly identified in two environments. The rest of ten QTLs were detected just in one environment, accounting for phenotypic variation of 2.81 to 9.30%. Four QTL clusters with common trait for FLL were found in this study. *QFLL-4A* was co-localized with *QFLA-4A* based on marker *Xwmc757*, with the Lumai 14-derived alleles simultaneously increasing FLL and FLA. *QFLL-1A* associated with *QFLA-1A* and *QCC*_*S3*_*-1A* were detected on chromosome 1A, and the favorable alleles were also derived from Lumai 14. The alleles of the QTL clusters formed by the remaining two QTLs for FLL (*QFLL-5D* and *QFLL-6A*) were from Shaanhan 8675, which were co-localized QTLs for FLW (*QFLW-5D* and *QFLW-6A*) and QTLs for FLA (*QFLA-5D* and *QFLA-6A*) on chromosomes 5D and 6A, respectively.

Compared with the above results, we found two QTLs, *QFLW-6A* for FLW and *QFLA-6A* for FLA, were detected in Lumai 14 / Jing 411 IL population in E2 environment and in Lumai 14 / Shaanhan 8675 population in E1 and E2 environments, respectively, suggesting that they were stable QTLs. And the two loci were linked with marker *Xwmc201*, indicating that they may be pleiotropic or tightly linked.

### Genetic effect analysis of *QFLW-6A*

Based on the above results, we found that the *QFLW-6A* was a stable QTL detected under E2 environment in Lumai 14 / Jing 411 IL population and under E1 and E2 environments in Lumai 14 / Shaanhan 8675 IL population. Therefore, it is necessary to analyze the genetic effect *QFLW-6A* on flag leaf width.

**Fig 2:**
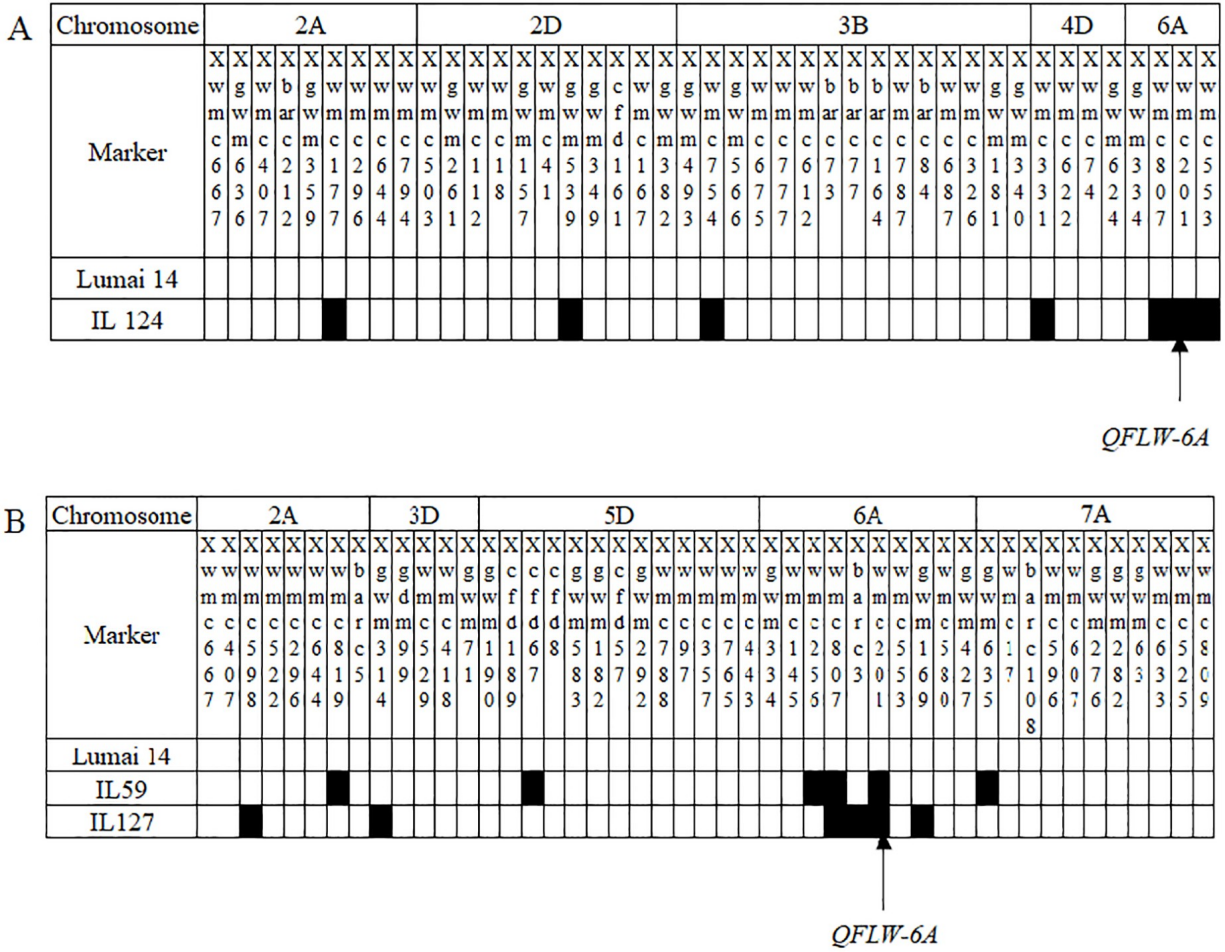
The distribution of introgression fragments of the near-isogenic lines in two IL populations. The chromosomes not shown were no substituted segments from donor. A: Lumai 14 / Jing 411 population; B: Lumai 14 / Shaanhan 8675 population.

Genome-wide scanning and QTL mapping found that 18 lines in Lumai 14 / Jing 411 population contained *QFLW-6A* with the favorable allele originating from Jing 411. The number of chromosomal fragments from donor (Jing 411) ranged from four to twenty-one in all 18 lines. Among them, IL 70 was introgressed four donor chromosomal fragments, while IL 18, 69 and 124 contained five donor fragments, respectively. On the other hand, the mean value of FLW in all lines with the locus *QFLW-6A* was higher than that of recipient parent (Lumai 14) in two environments. The FLW of IL 12 and 110 showed significant difference from Lumai 14 in E1. The FLW of IL 4, 8 and 31 showed significant difference compared with Lumai 14 in E2 environment. In particular, The FLW of IL 124 was significantly wider than Lumai 14 in both E1 and E2. It’s worth noting that IL 124 carried no other QTL controlling FLW than *QFLW-6A* (S1 Table, Fig 2A). Therefore, the IL 124 and their recurrent parent can be regarded as potential near-isogenic lines (NILs).

For Lumai 14 / Shaanhan 8675 IL population, *QFLW-6A* was repeatedly detected both in E1 and E2, with positive additive effect from Shaanhan 8675. Forty-four lines anchored this locus. Among of them, 11 lines (IL 2, 19, 59, 76, 77, 80, 104, 118, 126, 127 and 157) were substituted fragments from Shaanhan 8675 no more than five fragments. On the other hand, the mean FLW of all lines with *QFLW-6A* was wider than that of Lumai 14 in two environments. Compared with Lumai14, 17 lines (IL 42, 57, 58, 73, 78, 79, 86, 90, 91, 92, 94, 95, 102, 104, 125, 128 and 145) presented significantly wider FLW in E1, and three lines (IL 75, 80 and 126) did in E2. Especially, the FLW of ten lines (IL 3, 22, 24, 26, 59, 60, 74, 93, 127 and 151) was significantly wider than that of recipient parent in both E1 and E2 environments (S1 Table, Fig 2B). These results indicated *QFLW-6A* played an important role for increasing FLW. Because the IL 59 and 127 was introgressed five and four donor chromosomal fragments, respectively, on which no other QTL for FLW was detected, the IL 59 and 127 and their recurrent parent can be taken as potential NILs.

### Fine mapping for *QFLW-6A*

Through sequence alignment with wheat reference genome sequence (IWGSC RefSeq v1.0) published by International Wheat Genome Sequence Consortium (IWGSC), the physical location of the amplified fragment of SSR marker *Xwmc201* linked with *QFLW-6A* was found to be 574,283,851–574,283,613 bp on chromosome 6A. And 10 genes were found by further analysis of the genetic information in the range of 500 kb upstream and downstream (573,783,851–574,783,851 bp) of the fragment, including genes encoding F-box family protein and protein kinase family protein, etc (http://plants.ensembl.org/index.html) (Table 4).

**Table 4:** The predicted genes on the location of *QFLW-6A*.

Gene ID	Position (bp)	Genes Description
*TraesCS6A02G341000*	573,860,671–573,861,709	F-box family protein
*TraesCS6A02G341200*	574,220,464–574,221,965	F-box/RNI-like/FBD-like domains-containing protein
*TraesCS6A02G341300*	574,238,362–574,239,045	F-box domain containing protein-like
*TraesCS6A02G341400*	574,242,017–574,243,759	3-ketoacyl-CoA synthase
*TraesCS6A02G341500*	574,249,221–574,249,521	alpha-adaptin
*TraesCS6A02G341600*	574,378,180–574,386,049	GPI-anchored adhesin-like protein
*TraesCS6A02G341700*	574,472,578–574,479,877	RRP12-like protein
*TraesCS6A02G341800*	574,481,191–574,488,679	carboxyl-terminal peptidase, putative (DUF239)
*TraesCS6A02G341900*	574,489,301–574,493,373	Chaperone protein dnaJ
*TraesCS6A02G342000*	574,524,921–574,530,023	Protein kinase family protein

## Discussion

### Comparison with previous results

Flag leaf related-traits of wheat belong to quantitative traits, which have complex genetic basis and are greatly influenced by environment. Due to mapping QTL by different mapping population under different environments and using diversity of marker type, the results were difficult to replicate. Twenty-nine QTLs for flag leaf-related traits were identified in this study, only a few were completely consistent with previous results. For FLL, six QTLs were detected in this study. Among them, three QTLs, *QFLL-2A*, *QFLL-4A* and *QFLL-4B*, were associated with the SSR marker *Xwmc667*, *Xwmc757* and *Xwmc47*, respectively, which were reported at similar genetic regions [10, 43–45]. And *QFLL-4A* were detected under two environments in this study. *QFLL-6A* was also identified under two environments, which was located near the marker *Xwmc201* on chromosome 6A. *QFLL-5D* linked to the SSR marker *Xcfd189* in this study accorded with the QTL for FLL reported by Fan et al. [10], which was located the flanking interval *Xcfd189*-*Xgwm174*. For FLW, six QTLs were identified in this study. Among them, two QTLs, *QFLW-3B* and *QFLW-5D*, linked to the SSR marker *Xwmc754* and *Xcfd189* were previously found on the chromosomes 3B and 5D [7, 10]. *QFLW-6A* was consistently mapped on chromosome 6A in two IL populations, with contributing 6.96–9.28% to the phenotypic variance in different environments, and the positive allele of the QTL was originated from donors. Fan et al. [10] also found a QTL controlling FLW at the similar genetic region on this chromosome. It indicated that *QFLW-6A* was of importance to affect the FLW. For FLA, nine QTLs were detected in this study. Of them, *QFLA-1A*, *QFLA-2D*, *QFLA-4A* and *QFLA-6A* were distributed on chromosomes 1A, 2D, 4A and 6A, which have been proved on these chromosomes [7, 10]. It was worth mentioning that *QFLA-6A* was simultaneously in Lumai 14 / Jing 411 population under E2 and in Lumai 14 / Shaanhan 8675 population under E1 and E2 environments, with explaining 5.28%, 13.32% and 6.18% of the phenotypic variance, respectively. It was likely a stable QTL controlling FLA. Throughout all QTLs detected in the present, *QFLW-6A* and *QFLA-6A* were identified in both IL populations, and the positive alleles of individual QTL were also derived from donors, suggesting that they could be stable QTLs. And the two loci were linked with *Xwmc201* on chromosome 6A at the same time, which indicated that they may be pleiotropic or tightly linked QTL responsible for both traits. We detected eight QTLs for CC in this study. Of these QTLs, *QCC*_*S3*_*-1A* associated with *Xbarc148* and *QCC*_*S2*_*-3A* associated with *Xwmc24* were repeatedly detected under two environments. *QCC*_*S1*_*-1B*, *QCC*_*S2*_*-3A* and *QCC*_*S2*_*-3B* were identified at similar regions on chromosomes 1B, 3A and 3B by Shi et al. [34] and Zhang et al. [16].

### Relationship between flag leaf-related traits and yield-related traits in wheat

The flag leaf, as the main organ for photosynthesis during the reproductive period, is responsible for the regulating final plant growth and yield formation in cereal crops [2, 46]. So, reasonablely increasing flag leaf size and decreasing the rate of chlorophyll degradation during grain-filling period can improve the photosynthetic ability and promote to increase photosynthetic products, and finally achieve to enhance yield. Numerous studies have shown that chlorophyll content and morphological traits of the flag leave were corelated with yield-related traits in phenotype in cereals [7, 8, 24, 43, 47]. And QTLs controlling related traits were not uniformly distributed on chromosomes, but tended to be distributed in the same or adjacent regions of the same chromosome [48–50]. In barely, a QTL for FLL and a QTL for spike length were simultaneously associated with gene *HvFT2*. Feltus et al. [51] reported a QTL cluster for FLW and thousand-kernel weight in chromosome 3S in sorghum. Yue et al. [52] found that the QTL controlling flag leaf-related traits and QTL for yield-related traits were distributed on the same genic regions in rice. Two QTL clusters, chlorophyll content and yield and chlorophyll content, yield, heading date and flowering date, were identified on the flanking interval *Xwmc718*-*Xwmc262* of 4B chromosome and *Xbarc320*-*Xwmc215*-*Xgdm63* of 5D chromosome, respectively, using DH population in wheat [15]. In addition, QTL clusters controlling flag leaf-related traits and yield-related traits were also detected on chromosomes 1A, 1B, 2D, 4A, 4D, 5A, 5B, 6B, 6D, 7B and 7D of wheat [7–10, 53]. We found some regions that not only controlling flag leaf-related traits but also yield-related traits in the same population. For example, *FLW-3B*, *FLA-3B*, *QCC*_*S2*_*-3B* and *QCC*_*S3*_*-3B* were identified near the SSR marker *Xwmc754* on chromosome 3B in Lumai 14 / Jing 411 population, where the *QTgw-3B* for thousand-kernel weight was also mapped using the same population [41]. And the favorable alleles of these QTLs were from Jing 411 increasing these traits. Besides, a QTL for CC, *QCC*_*S2*_*-1A*, detected in this study and *QPh-1A* for plant height and *QGwp-1A* for grain weight per plant previously detected were associated with the marker *Xwmc24* [41], with the alleles from Lumai 14 increasing CC_S2_ and plant height, however, decreasing grain weight per plant. Another QTL for CC, *QCC*_*S2*_*-3A*, was shared the same marker *Xwmc11* on chromosome 3A with a QTL controlling thousand-kernel weight previously identified [41], with the Lumai 14-derived alleles increasing CC_S2_, but decreasing thousand-kernel weight. In Lumai 14 / Shaanhan 8675 population, *QFLL-6A*, *QFLW-6A* and *QFLA-6A* were located to the SSR marker *Xwmc201*, which has been proved to linked to QTLs for kernel morphology-related traits by Chen et al. [36]. It was worth noting that the positive alleles of these QTLs were originated from Shaanhan 8675. So this locus could be pleiotropism or closely linked QTL, which not only affected flag leaf size but also affected kernel size. As we all know, the FLA was closely related with photosynthetic production, the larger the leaf area, the more photosynthetic products accumulated, the larger the grains. Therefore, it is of great significance to further study this locus for high-yielding selection in wheat breeding. The flag leaf-related traits are one of the key factors affecting plant structure and yield, therefore, we can detect stable QTLs and develop reliable molecular markers through the further study of "active regions" with the same effect that is responsible for multiple elite traits, which not only can promote genetic improvement of plant stature and yield in cereal crops, but also may be transfer an excellent gene controlling multiple traits into a plant at a time to improve the efficiency of breeding.

### Breeding of near-isogenic line with *QFLW-6A*

ILs are constructed through introgressing chromosomal fragments from a donor parent into a recipient parent after multiple backcross and self-cross, the genotypes of all progenies in the population are very similar to those of the recurrent parent. Phenotypic differences between lines and the recipient parent can generally be attributed to substituted fragments from donor [36, 41]. Backcross introgression has been the most commonly used method for developing NILs for QTL studies [54–58]. In the present study, the target QTLs were tracked with SSR markers. It was found that four lines in Lumai 14 / Jing 411 population and 11 lines in Lumai 14 / Shaanhan 8675 population were introgressed no more than five fragments from donor, and one of these fragments carried *QFLW-6A*, with the alleles from donors increasing FLW. In terms of FLW, only IL 124 in Lumai 14 / Jing 411 population and IL 59 and 127 showed significant difference from recipient Lumai 14 in both E1 and E2 environment. And they were significantly wider FLW than Lumai 14. While, the FLW of the remaining lines was no significant difference or significant only in one environment compared with Lumai 14, which may be caused by interaction effect between *QFLW-6A* with other introgressed fragments. In addition, the three lines were contained no other QTL besides *QFLW-6A*. Therefore, they can be regarded as potential NILs and *QFLW-6A* can be used for further fine mapping of FLW by crossing and backcrossing with recipient Lumai 14.

### Prediction of candidate gene for *QFLW-6A*

A synteny analysis found *QFLW-6A* was near the 574,283,851–574,283,613 bp fragment. And 10 genes were found in the range of 500 kb upstream and downstream of the fragment (Table 4 and Fig 1). These genes involved in life activities such as cell cycle regulation, cell apoptosis, signal transduction, growth and development, as well as biochemical processes such as resistance to stresses [59–62], and so on. At present, we are not sure that which one is the candidate gene of this QTL. It needs to be further analyzed and proved by biotechnology.

## Conclusion

A total of six, six, nine and eight QTLs for FLL, FLW, FLA and CC were detected in two IL populations, respectively. Of them, *QFLW-6A* and *QFLA-6A* were detected under E2 in Lumai 14 / Jing 411 population and under both E1 and E2 in Lumai 14 / Shaanhan 8675 population. *QCC*_*S3*_*-1A* from Lumai 14 / Jing 411 population and *QCC*_*S2*_*-3A*, *QFLL-4A* and *QFLL-6A* from Lumai 14 / Shaanhan 8675 population were repeatedly identified under two environments. Besides, eight QTL clusters for flag leaf size and CC were identified in the two IL populations. On the other hand, three potential near-isogenic lines carried no other QTL controlling FLW than *QFLW-6A* were found. *QFLW-6A* had an important role for increasing the FLW, with the favorable allele stemmed from donors in both IL populations. Further, *QFLW-6A* was found to be near the 574,283,851–574,283,613 bp fragment on chromosome 6A. These results can lay a foundation for identification of candidate gene and map-based cloning and functional verification of the QTL.

## Supporting information

S1 Table Flag leaf width characteristics in wheat lines carrying introgressed donor chromosomal fragments at the *QFLW-6A* locus in two IL population. (XLSX)
